# The Blue Color of Water

**Published:** 1882-06

**Authors:** 


### THE BLUE COLOR OF WATER.

The *Photographic News* states that Mr. Aitken has been studying the blue color so characteristic of the Mediterranean and the Lake of Geneva, and his conclusions are embodied in a paper presented last month to the Royal Society. Mr. Aitken begins by saying that two solutions have been offered of this puzzling problem—the one explained the color as due to reflection of small suspended particles which did not reflect the lower rays of the spectrum, and the other that the color was the result of the absorbent action of the water itself upon the white light, before and after reflection of these particles. The latter theory Mr. Aitken holds to be the true one. The smaller the number of white reflecting particles the darker and greener the water appears to be. Mr. Aitken having been successful in turning the still green water of Lake Como into a bright blue by scattering finely divided chalk in the middle of the lake.

